# Supplementary material for: Self-Study-Based Informed Decision-Making Tool for Empowerment of Treatment Adherence Among Chronic Heart Failure Patients—A Pilot Study
Source: Healthcare (Basel). 2025 Mar 20;13(6):685. doi: 10.3390/healthcare13060685 (PMC11942012; doi:10.3390/healthcare13060685)
Supplement: Supplementary file 1 [file healthcare-13-00685-s001.zip › healthcare-3379199-supplementary.pdf]

**Supplementary Materials**

**Table S1** Scores of questionnaire statements

| | *Control group (n = 40)* | *Test group (n = 40)* | *p-value* |
|---|---|---|---|
| MEDIAN SUM OF THE POINTS | 22 (19–24) | 22 (19–24) | 0.65 |
| Statement 1: HF is a chronic life-long disease | 3.5 (3–4) | 4 (3–4) | 0.19 |
| Statement 2: Foundational drug treatment of HF disease intends patient's well-being | 4 (3–4) | 4 (4–4) | 0.22 |
| Statement 3: The combination of HF drugs delays progression of HF disease | 3 (3–4) | 4 (3–4) | 0.009 |
| Statement 4: Daily intake of HF drugs is consistent with the vision of HF | 3 (3–4) | 4 (3–4) | 0.004 |
| Statement 5: controls: understanding of different aspects of my heart disease; test group: all questions are answered* | 3 (2.5–4) | 1 (0–1.5) | <0.001 |
| Statement 6: Improved understanding the different aspects of disease | 2 (1–4) | 3 (2–4) | 0.03 |
| Statement 7: Empowerment to take an informed decision for adherence self-management | 3.5 (3–4) | 3 (2–4) | 0.28 |

Using the Likert scale each statement was graded for agreement (grades of agreement: 0=not at all, 1 = rather no, 2 = rather yes, 3 = yes, 4 = absolutely). Values provided represent the mean; the numbers in parenthesis represent the 25-75% percentile. * the original statement had asked for presence of new questions after tool exposure while the controls were asked for understanding of aspects of their heart disease; for comparison, the scoring in the test group was inversed.

**Table S2** Comments from the test group

| Questions | Comments |
|---|---|
| I suffer from a chronic illness that requires lifelong daily treatment. | "I don't want to hear the reality."<br>"Lifelong daily treatment?" |
| The medications I take are intended to act on and ensure my well-being on the long term. | "Why don't I have all the treatment of the presentation?"<br>"Better define their usefulness" |
| The combinations of the different treatments help to slow down disease progression. | "The treatment also improved my quality of life."<br>"Are we sure?"<br>"The treatment may also produce side effects."<br>"This aspect needs to be more developed." |
| Daily medications is in agreement with my vision of the disease. | "I don't have choice."<br>"The number of drugs is too much." |
| Following the presentation, I have new questions that remained unanswered. | 7 patients wanted to have more detail on the lifestyle.<br>5 patients wanted to have more detail on the progression of the disease and the lifestyle.<br>1 patient wanted to have more detail on the progression of the disease and the treatment.<br>1 patient wanted to have more detail on the treatment and lifestyle.<br>1 patient wanted to have more detail on the treatment |
| This presentation allowed me to improve my understanding of my disease. | "The presentation speaks about the symptoms and the medications but gives little information about the disease."<br>"I have good knowledge about my disease."<br>"I need more explication about my disease." |
| This presentation helps me to take decisions to manage my disease. | "The presentation is very useful."<br>"Additional explanations from the doctor/nurse are necessary." |
| General comments | "This presentation is surely useful for a patient at the beginning of the disease."<br>"My understanding of my disease was already clear before the presentation."<br>"Concerning the figures given, this is abrupt/scary."<br>"Which sport and what intensity? importance of weight?"<br>"Add indication on diet (salt), importance of weight and type of physical activity possible, follow up mood/psyche"<br>"The presentation impresses/worries and creates confusion; the presentation should rather be individualized. It should not replace the explanations of the medical profession, because it is more confusing/worrying than useful. Presence of too much information." |

**Table S3** Comments from the control group

| Questions | Comments |
|---|---|
| I suffer from a chronic illness that requires lifelong daily treatment. | "I don't have any treatment actually." |
| The medications I take are intended to act on and ensure my well-being on the long term. | "If treatment is reintroduced." |
| The combinations of the different treatments help to slow down disease progression. | "The treatment keeps you alive." |
| Daily medication is in agreement with my vision of the disease. | No comments |
| I understand the different aspects of my heart disease. | 2 patients wanted to have more detail about the treatment.<br>1 patient wanted to have more details about the natural evolution of the disease.<br>1 patient wanted to have more details about the reason for the occurrence, natural evolution and treatment of the disease and also the "red flags" to know about. |
| I would like more information about my heart disease. | 2 patients wanted to have more detail about the progression of the disease and treatment.<br>3 patients wanted to have more details about the treatment.<br>1 patient wanted to have more details about the progression of the disease, treatment and lifestyle |
| With my current knowledge, I feel capable of making a decision with my doctor regarding my treatment. | 7 patients think it is up to the doctor to make the decision. |
| General comments | No comments |

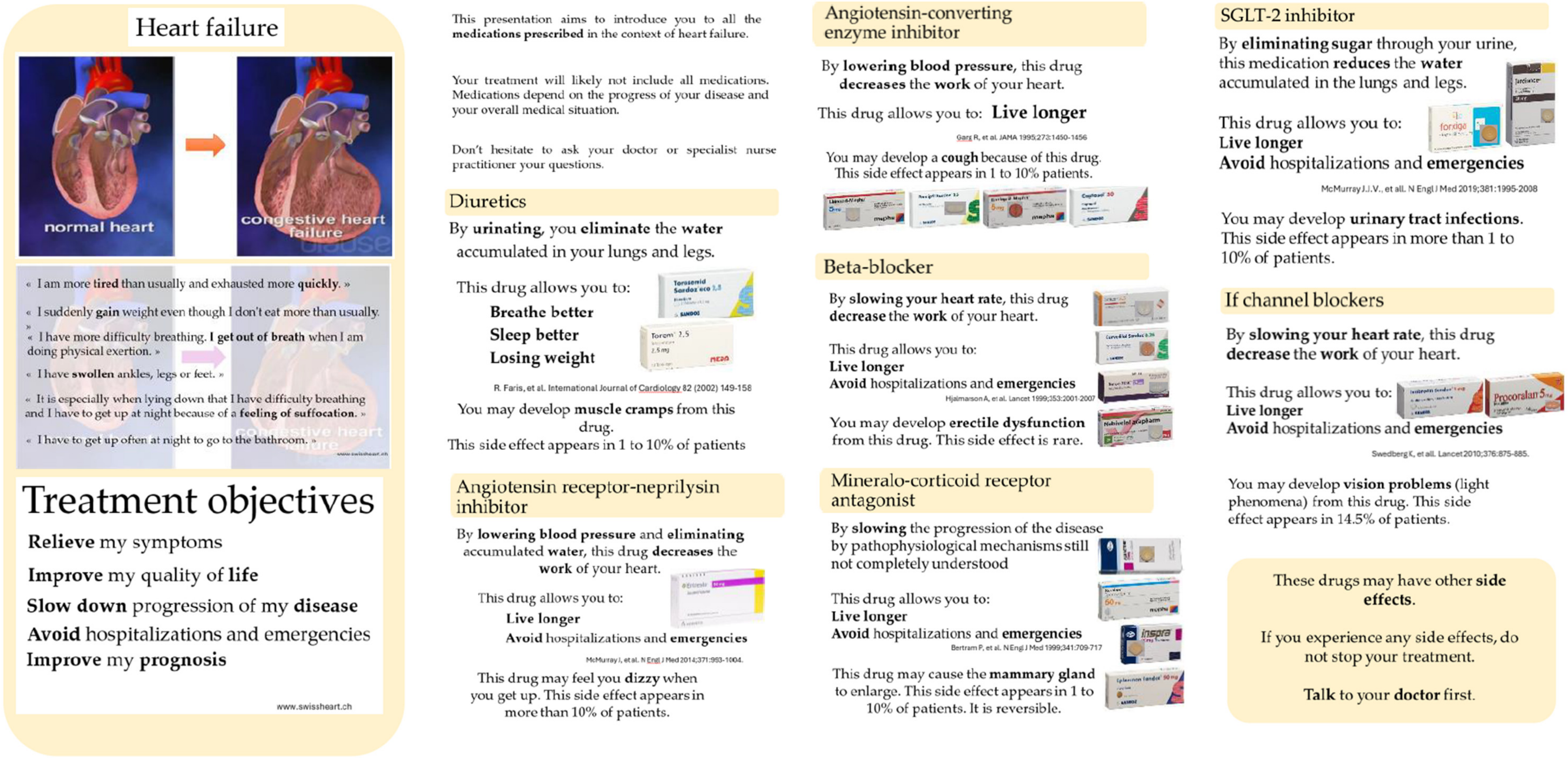


**Figure S1** Self-study informed-decision making tool

| Questions | 0 | 1 | 2 | 3 | 4 | Comments |
|---|---|---|---|---|---|---|
| I suffer from a chronic illness that requires lifelong daily treatment. | | | | | | |
| The medications I take are intended to act and ensure my well-being on the long term. | | | | | | |
| The combination of the different treatments helps to slow down the disease progression. | | | | | | |
| Daily medication is in agreement with my vision of the disease. | | | | | | |
| Following the presentation, I have new questions that remain unanswered. | | | | | | o Progression of the disease<br>o Treatment<br>o Lifestyle (physical activity, food) |
| This presentation allowed me to improve my understanding of my illness. | | | | | | |
| This presentation helps me to take decisions to manage my disease. | | | | | | |

(**a**)

| Questions | 0 | 1 | 2 | 3 | 4 | Comments |
|---|---|---|---|---|---|---|
| I suffer from a chronic illness that requires lifelong daily treatment. | | | | | | |
| The medications I take are intended to act on and ensure my well-being on the long term. | | | | | | |
| The combination of the different treatments helps to slow down the disease progression. | | | | | | |
| Daily medication is in agreement with my vision of the disease. | | | | | | |
| I understand the different aspects of my heart disease. | | | | | | o Reason for the occurence of the disease<br>o Natural evolution of the disease<br>o Treatment (drugs, devices, lifestyle)<br>o Reds flags (weigth gain, shortness of the breath, edema, dizziness) |
| I would like more information about my heart disease. | | | | | | o Progression of the disease<br>o Treatment<br>o Lifestyle (physical activity, food)<br>o Others: _______ |
| With my current knowledge, I feel capable of making a decision with my doctor regarding my treatment. | | | | | | o I would like to explore the following aspects in more detail: _____<br>o I think it is up to my doctor to make the decision. |

(**b**)

**Figure S2** (a)Interview protocol of the test group (b) Interview protocol of the control group. 0 = Not at all. 1 = Rather no. 2 = Rather yes. 3 = Yes. 4 = Totally

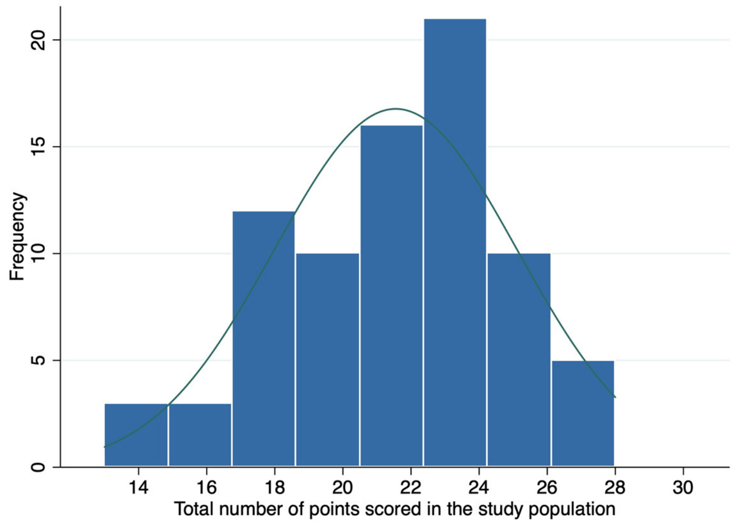


(**a**)

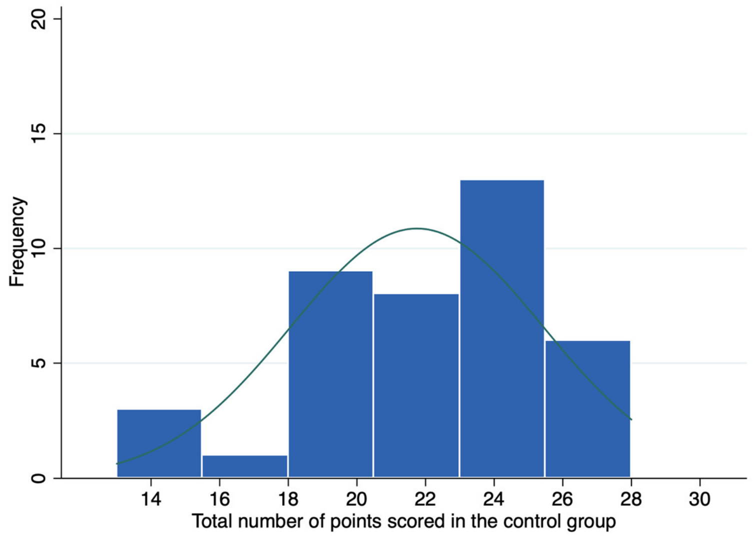


(**b**)

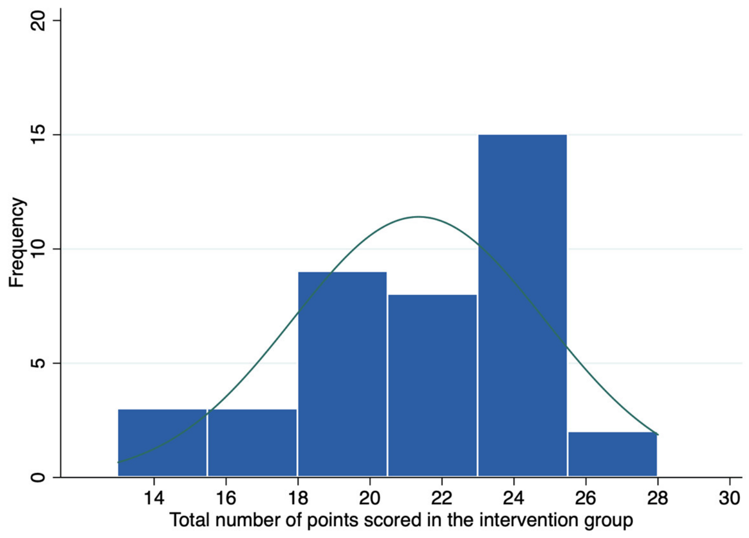


(**c**)

**Figure S3**. (a) Distribution of individual patient's total score in all study participants (b) Distribution of individual patient's total score in the control group (c) Distribution of individual patient's total score in the intervention group
